# P36 Crohn’s-associated inflammatory arthritis treated successfully with upadacitinib

**DOI:** 10.1093/rap/rkac067.036

**Published:** 2022-09-28

**Authors:** James Carroll, Fraser Cummings, Christopher Holroyd

**Affiliations:** Rheumatology, Poole Hospital, Poole, United Kingdom; Gastroenterology, Southampton General Hospital, Southampton, United Kingdom; Rheumatology, Southampton General Hospital, Southampton, United Kingdom

## Abstract

**Introduction/Background:**

Enteropathic arthritis is inflammatory arthritis which is associated with inflammatory bowel disease such as Crohn’s disease and ulcerative colitis. The optimal treatment for patients is one which is effective for both arthritis and bowel disease. First line treatments include DMARDs such as methotrexate and sulfasalazine. Second line options include anti-TNF biologics such as infliximab and adalimumab, and the anti-IL-12/23 biologic ustekinumab. There is currently little guidance on choice of treatment if these options fail. We present a case of Crohn’s-associated inflammatory arthritis which did not respond to two anti-TNFs but was successfully treated with the JAK inhibitor upadacinitib.

**Description/Method:**

A 30-year-old male was referred to gastroenterology with a 4 month history of diarrhoea and weight loss. His faecal calprotectin was raised at 982µg/g. He had a history of seronegative inflammatory arthritis diagnosed 5 years previously. He had been treated initially with methotrexate and sulfasalazine, which were discontinued due to side effects and inefficacy, and his arthritis was subsequently well-controlled on etanercept for the last year.

Colonoscopy showed patchy colitis at the rectum which was continuous in the transverse and ascending colon and caecum. The appearances and mucosal biopsy were consistent with moderately active Crohn’s colitis. In view of this, etanercept was switched to adalimumab, which was administered using the accelerated loading regimen used in IBD.

Five weeks after switching the patient developed a flare of arthritis with knee and ankle inflammation and CRP 30. He was given oral steroids and reviewed in a combined rheumatology/gastroenterology clinic. The frequency of adalimumab was increased to 40mg weekly and subcutaneous methotrexate 10mg weekly was introduced.

Despite this, the patient’s arthritis remained active, although with some improvement in his bowel symptoms and a fall in faecal calprotectin to 117µg/g. Adalimumab was switched to certolizumab and methotrexate increased to 15mg weekly with intra-muscular steroids. On certolizumab he developed progressively more active arthritis with CRP 83, bilateral ankle inflammation and large knee effusions requiring aspiration and steroid injection.

After 8 weeks of certolizumab, there was no improvement and the decision was made to switch to upadacitinib 15mg daily. Within 4 weeks of treatment with upadacitinib he noticed a significant improvement in his arthritis and after 12 weeks was in clinical remission with normal CRP and faecal calprotectin. Methotrexate was later discontinued due to nausea. Over 12 months later the patient remains in remission for both bowel disease and arthritis on upadacitinib monotherapy.

**Discussion/Results:**

In enteropathic arthritis, joint symptoms may precede or follow bowel disease. Etanercept, a recombinant TNF-receptor fusion protein, is not an effective treatment for IBD. Indeed, there are case reports and observational data to suggest that etanercept may be associated with an increased risk of developing IBD in some patients. In our case the patient developed Crohn’s disease while taking etanercept and the switch to adalimumab precipitated a flare of his arthritis which was not controlled by treatment intensification. The failure of two anti-TNF biologics, adalimumab and certolizumab, may point to the activation of alternative cytokine inflammatory pathways as the driver of the patient’s disease.

Our patient was managed in a combined rheumatology/gastroenterology clinic which enabled an inter-specialty approach to managing both aspects of his disease. After the failure of certolizumub we made the decision to trial upadacitinib, a selective JAK1 inhibitor, owing to our experience of its effectiveness in rheumatoid and psoriatic arthritis, and its promising results in Phase 3 trials in Crohn’s disease. We considered ustekinumab but opted against it as in our experience it may be suboptimal in patients in whom arthritis is the most troublesome symptom. Upadacitinib worked quickly and effectively to control our patient’s arthritis alongside his bowel disease and was well tolerated.

There are currently limited treatments which are effective for both IBD and arthritis, and there is little guidance on treatment strategies in enteropathic arthritis if anti-TNF and ustekinumab are unsuccessful. Tofacitinib, a JAK1/3 inhibitor, is licensed for rheumatoid and psoriatic arthritis and ulcerative colitis, but is not effective for Crohn’s disease. Upadacitinib is undergoing Phase 3 trials in Crohn’s disease and our case highlights its potential role in the treatment of patients with Crohn’s disease and inflammatory arthritis in whom anti-TNF therapy is unsuccessful.

**Key learning points/Conclusion:**

1. Etanercept is not an effective treatment for inflammatory bowel disease and may necessitate a change to another anti-TNF biologic such as infliximab or adalimumab.

2. Upadacitinib, a selective JAK1 inhibitor, was successfully used in our case to treat both arthritis and bowel disease. Although this was just one case, the results of Phase 3 trials suggest a promising role for upadacitinib in Crohn’s disease and it is already used in the treatment of rheumatoid arthritis, psoriatic arthritis and ankylosing spondylitis.

3. Patients with enteropathic arthritis may be best managed in a specialist combined gastroenterology and rheumatology clinic where a holistic approach to the management of both aspects of their disease can be undertaken.

